# Potential Application of Photosensitizers With High-Z Elements for Synergic Cancer Therapy

**DOI:** 10.3389/fphar.2022.921729

**Published:** 2022-06-28

**Authors:** Paromita Sarbadhikary, Blassan P. George, Heidi Abrahamse

**Affiliations:** Laser Research Centre, Faculty of Health Sciences, University of Johannesburg, Johannesburg, South Africa

**Keywords:** chemotherapy, chlorophyll derivatives, heavy atoms, metals, photodynamic therapy, tetrapyrroles, X-ray photon activation therapy

## Abstract

The presence of heavy elements in photosensitizers (PS) strongly influences their electronic and photophysical properties, and hence, conjugation of PS with a suitable element is regarded as a potential strategy to improve their photodynamic properties. Moreover, PS conjugated to metal ion or metal complex and heavy atoms such as halogen have attracted considerable attention as promising agents for multimodal or synergistic cancer therapy. These tetrapyrrole compounds depending on the type and nature of the inorganic elements have been explored for photodynamic therapy (PDT), chemotherapy, X-ray photon activation therapy (PAT), and radiotherapy. Particularly, the combination of metal-based PS and X-ray irradiation has been investigated as a promising novel approach for treating deep-seated tumors, which in the case of PDT is a major limitation due to low light penetration in tissue. This review will summarize the present status of evidence on the effect of insertion of metal or halogen on the photophysical properties of PS and the effectiveness of various metal and halogenated PS investigated for PDT, chemotherapy, and PAT as mono and/or combination therapy.

## Introduction

Even with the advancement in treatment modalities and diagnosis, cancer remains the deadliest disease worldwide, claiming almost 10 million lives alongside the incidence of 19.3 million new cancer cases, only in 2020. The recent cancer statistics show the rapid and worrisome increase in the burden of cancer incidence and mortality with an estimated trajectory of 28.4 million cases by 2040, almost ∼47% rise from 2020, due to increasing risk factors associated with socioeconomic developments (Sung et al., 2021). The conventional treatment modalities used to treat cancer includes surgery, radiotherapy, and chemotherapy dependent on different stages, metastatic property of cancer with systemic or localized treatment outcomes. Over the past 25 years, research studies in cancer therapeutics have not only led to significant developments in conventional therapies but also provided some novel approaches to cancer treatments, which holds considerable potential to treat cancer with better efficacy and improved outcome (Charmsaz et al., 2019; Pucci et al., 2019).

**FIGURE 1:**
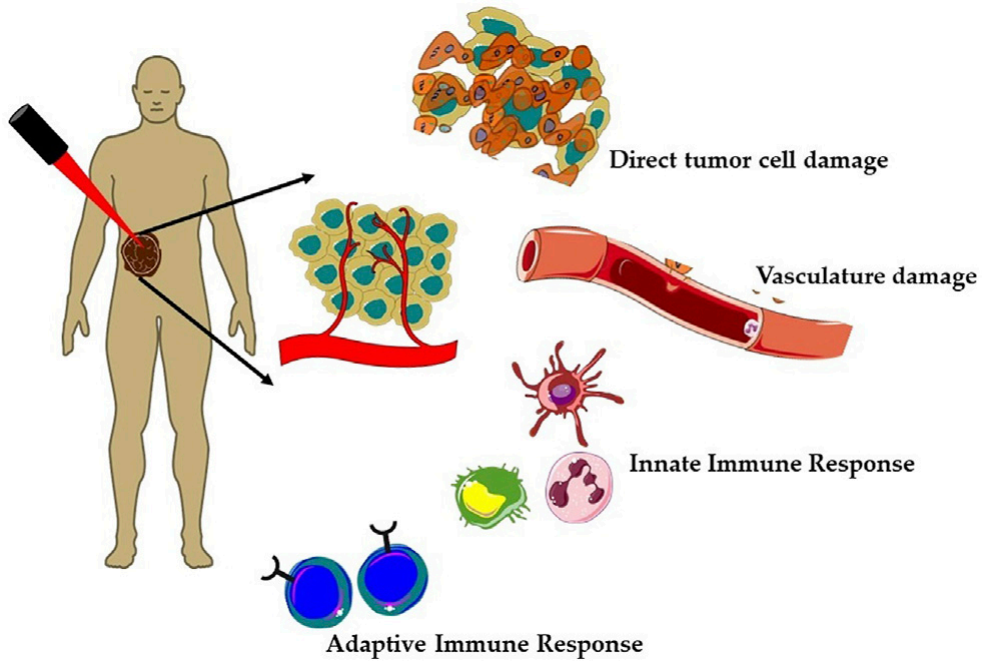
Mechanisms of action of photodynamic therapy (PDT) on solid tumors. Generation of reactive oxygen species (ROS) during PDT induces a plethora of cellular effects; direct tumor cell death and tumor vasculature damage and immune responses, that is, activation of both innate and adaptive immune responses against tumor mass.

Over the past few decades, clinically approved photodynamic therapy (PDT) has emerged as a promising alternative therapeutic modality for superficial cancers and as adjuvant therapy among the unconventional therapies (Baskaran et al., 2018; Alsaab et al., 2020; Li et al., 2020; Hu et al., 2021). Currently, PDT is clinically approved for the treatment of several types of cancer such as breast cancer, gynecological, intraocular, brain, head and neck tumors, colorectal cancer, cutaneous malignancies, intraperitoneal tumors, mesothelioma, cholangiocarcinoma, and pancreatic cancer (Huang, 2005; Allison and Sibata, 2010; Fayter et al., 2010; Li et al., 2020). Mechanistically, PDT is a photochemistry-based treatment approach that utilizes a photoactivable drug referred as photosensitizer (PS) followed by irradiation with red or near-infrared light to induce tumor damage *via* the generation of reactive oxygen species (ROS) (Agostinis et al., 2011; Li et al., 2020). PDT offers several advantages such as selective killing of cancer cells, lower risk of developing resistance, and less systemic toxicity as compared to chemotherapy and radiotherapy (Olivo et al., 2010). The tumor selectivity in PDT is achieved in a dual manner, first using a photosensitizer (PS), which preferentially localizes in malignant tissue and then confining the irradiation to the affected site using an intense beam of light either from non-coherent light sources (e.g., arc lamps) or laser/fiber optic systems. As represented in Figure 1, tumor regression induced by PDT is a consequence of complex mechanisms involving direct tumor destruction, tumor vasculature damage, and antitumor immune response (Agostinis et al., 2011; Abrahamse and Hamblin, 2016; van Straten et al., 2017; Li et al., 2020). The effectiveness of PDT has been demonstrated in several clinical studies including cancers, which failed to respond to traditional treatments. However, PDT also suffers from certain major limitations; first, due to poor penetration depth of light in tissue, which restricts the application of PDT as only palliative surface treatment (van Straten et al., 2017). Second, PDT efficacy depends heavily on the availability of O_2_ in the tissue environment and thus it decreases significantly in solid tumors, due to the hypoxic conditions (Huang et al., 2008; van Straten et al., 2017; Pucelik et al., 2020). Thus, as every single anticancer treatment modality suffers from their inherent limitations and with increased incidences of drug resistant cancers and their relapse, much research effort has been dedicated toward the development of novel multitherapeutic conjugates for synergic therapeutics (Karges, 2022). Several studies have reported the importance and effectiveness of combination of PDT with other therapies like chemotherapy, immunotherapy, photothermal therapy, radiotherapy, and sonodynamic therapy (Postiglione et al., 2011; Brodin et al., 2015; Hwang et al., 2018; Zhang and Li, 2018; Zheng et al., 2020; Karges, 2022).

### Photochemistry and Photophysics of PDT and Photosensitizers

**FIGURE 2:**
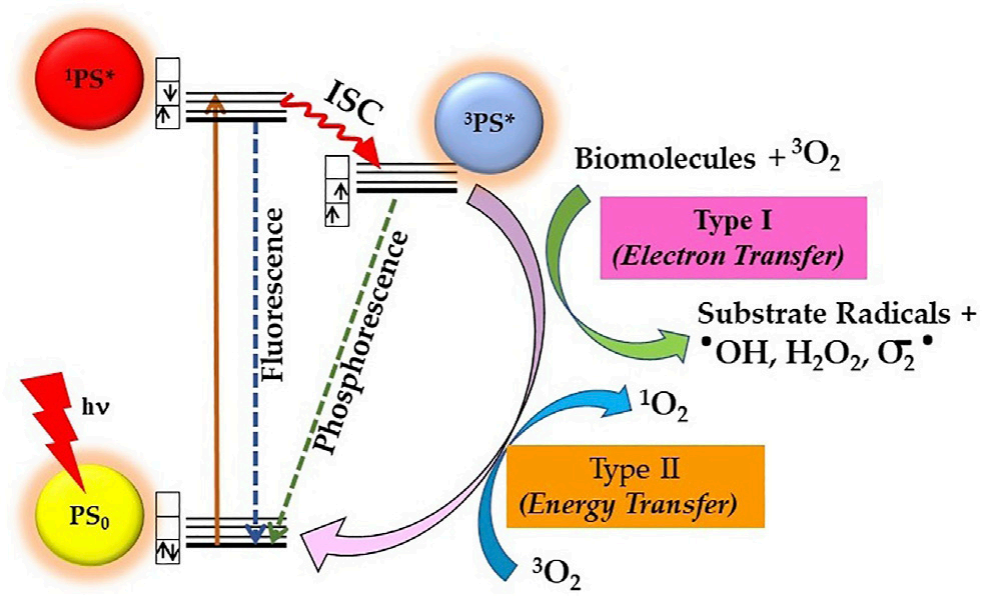
Principle of photodynamic therapy (PDT) represented by a modified Jablonski diagram: Excitation of ground state PS0 into its excited singlet state ^1^PS* occurs by light energy of appropriate wavelength. Spin-forbidden transition from ^1^PS* to excited triplet ^3^PS* occurs through intersystem crossing (ISC), whereby ^3^PS* roots into reactive oxygen species (ROS) generation *via* Type I and/or Type II photochemical reactions.

Mechanistically, the photosensitization process involves both photophysical and photochemical processes, whereby PS upon absorption of light energy transfers it to nearby substrate molecules to generate ROS. As shown in Figure 2, during the photophysical process, the PS molecule after absorption of light is excited from its ground state (PS_0_) to its short-lived (nanoseconds) excited singlet state (^1^PS*). The ^1^PS* state either directly decay back to its ground state releasing energy in the form of a photon emission (fluorescence) or heat (non-radiative decay) or converted into a long lived and chemically more reactive excited triplet state ^3^PS* *via* intersystem crossing (ISC). This ^3^PS* can either decay to the ground state radiationlessly or undergo photochemical reactions *via* type I and/or type II mechanisms. Type I process involves hydrogen-atom abstraction or electron transfer process from ^3^PS* to a nearby biological substrate or O_2_ molecules, resulting in the formation of free radicals and radical ions. Furthermore, the highly reactive-free radicals of a substrate molecule can readily interact with O_2_ to either generate ROS such as superoxide anions (O_2_•-) or hydroxyl radicals (•OH) or can cause irreparable biological damage. In type II pathway, ^3^PS* transfer its energy to O_2_ to form highly reactive singlet oxygen (^1^O_2_). The resulting highly reactive ROS with short lifetime eventually oxidizes biomolecules such as nucleic acids, amino acids, or unsaturated lipids within a radius of about 100 nm, leading to cell death. Both processes occur simultaneously, but the prevalence between them depends upon PS property, availability of O_2_, and surrounding environment (Castano et al., 2004; Josefsen and Boyle, 2008; Bacellar et al., 2015; Abrahamse and Hamblin, 2016). In general, PDT efficacy of majority of PS is *via* ^1^O_2_ generated through type II mechanism (Castano et al., 2005; Karges, 2022).

**FIGURE 3:**
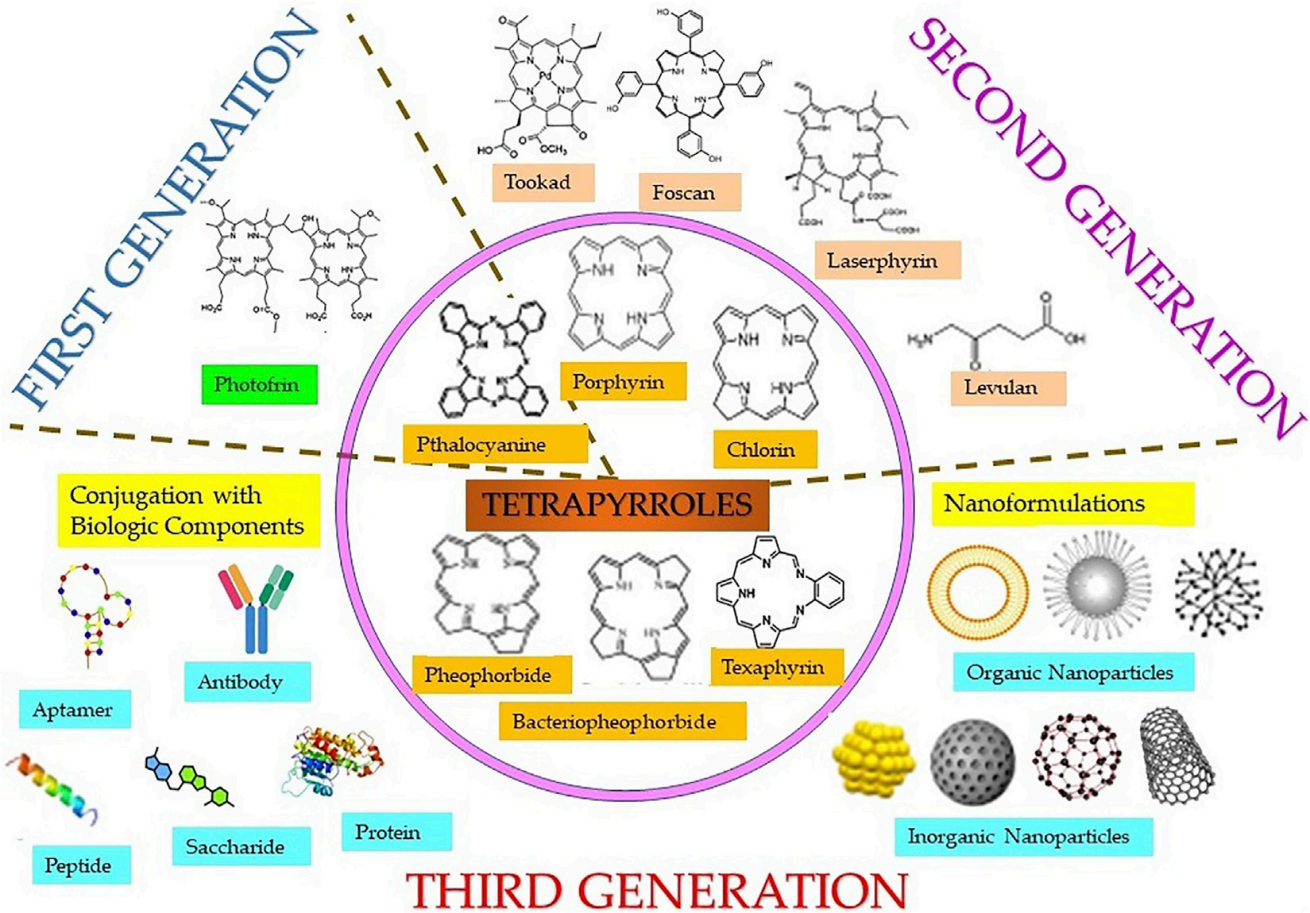
Classification of different classes (inner circle) of tetrapyrrole photosensitizers and different generations of photosensitizers including first, second, and third generation (in the form of conjugates linked with targeting biologic moieties and nanoparticles).

Both natural and synthetic PS are being used in cancer PDT, among which majority are tetrapyrrole macrocyclic structure such as porphyrins (including texaphyrins), chlorins, bacteriochlorins, and phthalocyanines (Figure 3). These tetrapyrrolic PS have extended π-electron systems, which are responsible for their unique photophysical and photochemical properties. PS have been classified as first, second, or third generation as shown in Figure 3 (Stacey and Pope, 2013; Karges, 2022). Discovery of hematoporphyrin (Hp) in 1841 and the clinical applications of its derivatives (HpD) by Dr. Thomas Dougherty and his colleagues lead to the first-generation PS. As compared to Hp, HpD showed better tumor tissue selectivity, with less skin photosensitivity. HpD is commercially available under the trade name Photofrin and is basically a mixture of monomers, dimers, and oligomers of Hp. In 1993, Photofrin became the first PDT reagent, to be clinically approved for bladder cancer treatment and later approved for the treatment of esophageal cancer, bladder cancer, and gastric cancer by regulatory authorities from different countries throughout the world. Despite its wide applications in PDT, it suffers from several drawbacks like low chemical purity, poor tissue penetration, and high skin hypersensitivity for several weeks, which limits its clinical applications. These disadvantages of the first-generation PS imposed the necessity of investigating better PS and initiated the development of the second-generation PS in late 1980s. The second-generation PS offer several advantages such as better penetration into deeply located tissues due to maximum absorption wavelength in the therapeutic window (650–800 nm), high singlet oxygen or ROS generation efficacy, and higher chemical purity, along with fewer side effects a consequence of relativity preferential tumor accumulation and faster clearance from body. Currently, the second-generation PS comprise porphyrin precursors, and cyclic tetrapyrrole ring compounds synthetic photosensitizers, whose chemical modifications distinguish the different groups such as porphyrin, chlorins, 5-aminolevulinic acid, benzoporphyrin derivatives, phthalocyanines, naphthalocyanines, texaphyrins, thiopurine derivatives, and bacteriochlorin analogs. Many of these second-generation PS have been approved for several different cancers, but there are disadvantages in this generation PS like poor solubility in water, which significantly limits their intravenous administration and displays suboptimal bioavailability in malignant tissues compels the search for novel PS delivery strategies. This includes conjugation of second-generation PS with biological targeting components to promote their selective localization and accumulation at the tumor site along with reducing the damage to surrounding, healthy tissues, and thus encompasses the advanced third-generation class of PS. Some of the commonly used targeting moiety being used for conjugations includes target receptor, monosaccharides, peptides, proteins, LDL lipoprotein, monoclonal antibodies directed to the specific antigen of cancer cell, tumor surface markers such as growth factor receptors, transferrin receptors, or hormones (e.g., insulin). Furthermore, with the introduction of nanotechnology, nanoformulations of PS, which consist in second-generation PS conjugated or encapsulated within NPs have also emerged as the third-generation PS. Nano-PS offer several advantages over free second-generation PS such as high stability, high loading or conjugation efficiency, adjustable size, optical properties, easy surface functionalization, slow degradation, long cycle time, high biocompatibility, and resistant to decomposition in biological applications, which allows tumor targeting, delivery, and controllable release of PS for improved PDT efficacy (Lismont et al., 2017; Kwiatkowski et al., 2018).

Due to enhanced photophysical characteristics of second-generation PS such as high absorption coefficient in the red wavelength region (>630 nm), higher yield of long-lived ^3^PS*, and efficient ^1^O_2_ generation, they offer better PDT effectiveness (Szaciłowski et al., 2005; Ormond and Freeman, 2013; Abdel-kader, 2016). However, majority of these PS suffer from several drawbacks, including poor photostability and water solubility, tedious synthesis/purification, alongside with poor cancer selectivity slow and body clearance causing photosensitivity (Stacey and Pope, 2013; Karges, 2022). Thus, more attention is focused on improvement and development of advanced smart and novel PS suitable for clinical applications. One such strategy that has been explored over the past few years is the introduction of heavy atom or high Z elements into PS to their improved photostability and photophysical and photochemical properties compared to their free PS counterpart. More importantly, this approach of designing efficient PS enhances ISC by intensifying the coupling of the singlet and triplet states of PS or decreasing their relative energy gap. This results into efficient production of triplet excited states, which ultimately enhances the ROS generation efficiencies important for effective PDT outcome (Nguyen et al., 2021; Pham et al., 2021). In this review, we present the effect of introduction of metal and heavy elements on photophysical properties of tetrapyrrolic PS and summarize their applications for effective PDT and in combination with other therapies like chemotherapy and radiotherapy. The final section discusses the challenges that are needed to be addressed for the development of heavy-atom PS for clinical applications. Importantly, this review attempted to provide insights of the molecular design approaches of heavy atom PS for investigating their important role in PDT and other potential treatment modalities.

### Effect of Metal and Heavy Elements on Photophysical and Photodynamic Properties of PS

**FIGURE 4:**
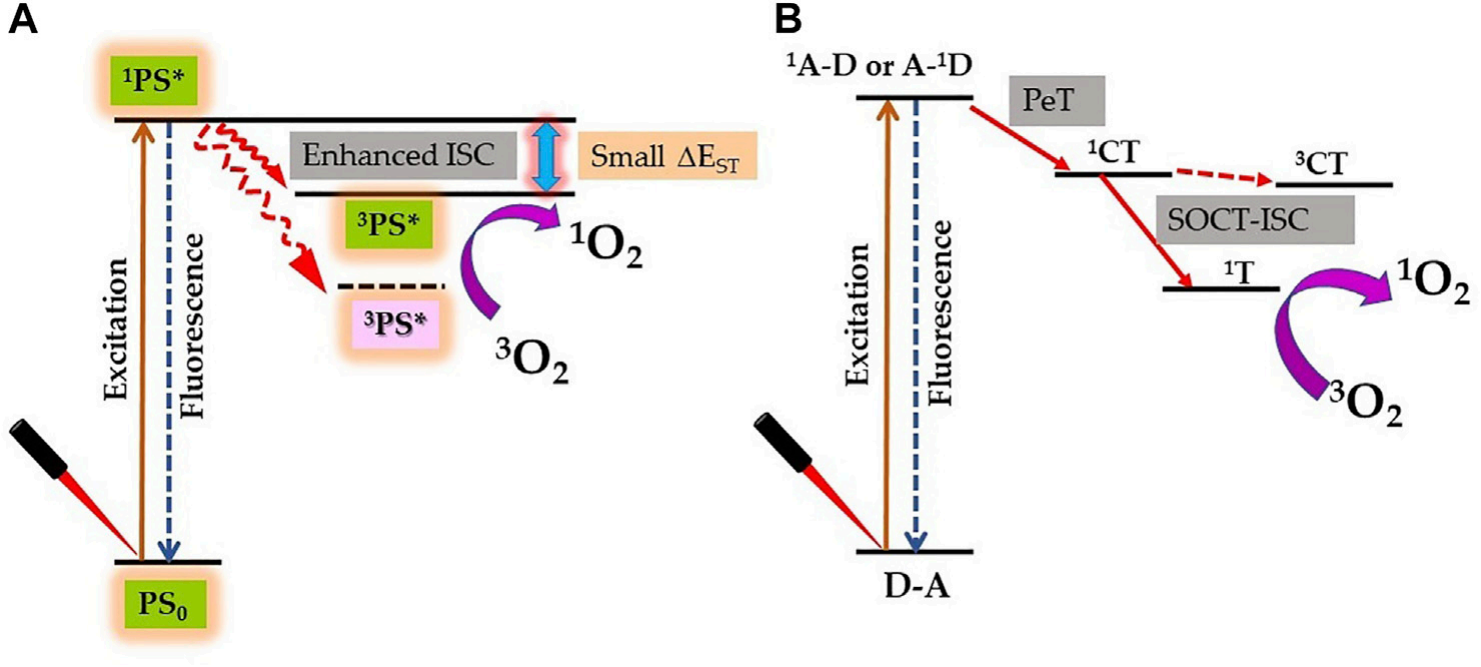
Proposed strategy for enhanced singlet oxygen (^1^O_2_) generation by the heavy atom effect. **(A)** Reducing the singlet–triplet energy gap (ΔE_ST_). **(B)** Spin–orbit charge transfer intersystem crossing (SOCT-ISC) mechanism by electron donor‐acceptor (D–A) pair [^1^A–D: singlet state of an acceptor, A–^1^D: singlet state of a donor; PeT: photoinduced electron transfer; ^1^CT: singlet charge separated state; ^3^CT: triplet charge separated state; and ^1^T: triplet state].

The lifetime and quantum yield of ^3^PS* correlate with its ability to produce adequate levels of ROS and therapeutic outcomes; hence, these are important requirements of potential PS (Mehraban and Freeman, 2015). Due to the heavy-atom effect, the attachment of heavy element to PS exerts a significant effect on its excited states and therefore influences its photosensitization efficiency. In a recent review, authors have summarized the molecular design approaches of synthesis of heavy atom non-porphyrinoid PS based on: singlet−triplet energy gap reduction (Figure 4A), spin−orbit charge-transfer ISC (SOCT-ISC) (Figure 4B), twisted π-conjugation system-induced ISC, thionation of carbonyl groups of conventional fluorophores, and radical-enhanced ISC (Nguyen et al., 2021). Here, we will emphasize the heavy-atom effect on tetrapyrrolic PS, as although being natural compounds they induce less systemic toxicity; however, their designing and synthesis are bit challenging due to their robust and stable molecular structure.

As a general rule, introduction of a heavy atom, such as a halogen or transition metal to the tetrapyrrolic structure promotes ^1^PS* → ^3^PS* transition by spin–orbit perturbation/coupling (SOC) and thus improves the ROS generation yield (Solov’ev and Borisevich, 2005; Mehraban and Freeman, 2015). In general, SOC is the relativistic interaction between electrons’ spin and its orbital motion around the nucleus. SOC causes a shift in the electron׳s atomic energy levels of an electron moving in the finite electric field of the nucleus, occurring due to the electromagnetic interaction between the spin of the electron and the electric field. For efficient ISC of PS, the energy and the total angular momentum (orbital and spin) have to be conserved. Thus, the process of SOC usually combines two spin states and also provides a means of conserving the total angular momentum. Thus, the heavy atom effect involves overlap between the molecular orbital of the tetrapyrrolic PS with the atomic orbitals of the perturbing large atomic number atoms, which can induce strong SOC, and results into enhancement in ISC. More mechanistically, the heavy atom effect is actually a nuclear-charge effect where an electron moving in the vicinity of a nucleus with positive charge Z will be accelerated to relativistic velocity, resulting into strong coupling between the spin and the orbital magnetic momentum. Therefore, the heavy atom effect increases with the atomic number Z of the atom as Z^4^ (Solov’ev and Borisevich, 2005; Marian, 2012, 2020; Zhao et al., 2013; Nguyen et al., 2021)

The majority of metal complex of tetrapyrrole compounds reported so far contain metal ion in the center of the macrocyclic ring. This is due to the fact that the macrocyclic ring with four nitrogen atoms at its central cavity acts as a tetradentate ligand for various metal ions. Photosensitization property of the heavy atom PS is influenced by the nature of the central metal ion bound to the macrocyclic ring. In general, diamagnetic metals such as Zn^+2^, Pd^+2^, In^+2^, Sn^+^4, and Lu^+3^ improve the ^3^PS* quantum yield whereas, paramagnetic ions like Mn^+3^, Fe^+3^, Co^+2^, Ni^+2^, and Cu^+2^ conjugated with tetrapyrroles reduce the ^3^PS* lifetime by deactivating the ^3^PS*. For example, insertion of Zn in tetraphenylporphyrin (TPP) showed an increase in the ^3^PS* quantum yields (Φ_T_) from 0.73 to 0.86, without any effect on ^3^PS* lifetime (τ_T_) (>10 μs), whereas insertion of Pd led to an increase in both and Φ_T_ and τ_T_ (>50 μs). For chlorin e6 (Ce6), insertion of Sn leads to increase in τ_T_ (240 μs) as compared to free Ce6 (50 μs), while for Cu complexes of TPP, meso-tetrakis (4-sulfonatophenyl) porphyrin (TPPS), and chlorin e4 (Ce4) τ_T_ is negligible (Gorman et al., 2004; O’Connor et al., 2009; Abdel-kader, 2016; Dąbrowski et al., 2016). Zn complex of picolylamine porphyrin exhibited Φ_T_ of ∼0.97, as compared to its freebase porphyrin derivative (0.64) (Marydasan et al., 2013). Chlorophyll molecule with Mg^2+^, Zn^2+^, and Cu^2+^ have τ_T_ of 209 ns, 199 ns, and 67 ns, respectively, as compared to metal-free pheophytin a with τ_T_ of 154 ns (Küpper et al., 2002).

τ_T_ of PS plays a significant role in induced phototoxicity, thus as compared to freebase counterpart, diamagnetic and heavy metal complexes of porphyrins, chlorins, and bacteriochlorins exhibit better PDT efficacy, due to higher efficiency to generate ROS. Whereas the paramagnetic metal complexes are usually photodynamically inactive. A systemic study carried out by Ando et al. (1993)showed that 2,4-bis (I -decyloxyethyl)-deuteroporphyrinyl-6,-7-bisaspartic acid and its Zn, Ga, In, and Sn complexes with longer (>1 ms) τ_T_ exhibited significant phototoxicity, while its metal complexes with Mn, Cu, Ni, and Fe, having short (<0.01 ms) τ_T_, showed negligible-to-no phototoxicity. Zn(II), In(III), and Ni(II) complexes of methyl pyropheophorbide-a were synthesized and among these analogs the In(III) complex showed the best PDT efficacy, while the Ni(II) complexes because of its inability to produce ROS did not show any PDT efficacy (Chen et al., 2005). Also, Fe (III) complex of meso-tetrakis (carboxyl) porphyrin meso-tetrakis (carboxyl) porphyrin did not lead to any phototoxicity against cancer cells (Shi et al., 2017). In another study, Pd(II) and Pt(II) complexes of chlorins showed strong phototoxicity in cancer cells in comparison to its freebase chlorin (Obata et al., 2009). The *in vitro* phototoxicity study with meso-tetrakis (4-sulfonatophenyl) porphyrin (TPPS4) and its metal complexes with Zn, Pd, and Mg, showed efficient ROS generation capability by ZnTPPS4 as compared to other PS, thus represented the most effective PS (Malina et al., 2016). However, unreasonably, phototoxicity induced by non-metalated bacteriochlorophyll and its Pd complex was almost the same, whereas its complexes with Cu, Zn, and Mn were photodynamically inactive (Brandis et al., 2005). Among the Ni, Cu, and Zn complexes of ^131^I labeled methyl 3-devinyl-3-{1′-(benzyloxy) ethyl} pheophorbide-a complexes, investigated for their photodynamic effectiveness against cancer cell lines, only the Zn complex was shown to be the most efficient PS. This complex effectively induced significant phototoxicity at a very low concentration of 1.5 μM and a light dose of 20 J/cm^2^, while the Ni and Cu complexes were able to induce ∼50% cell death at a very high concentration of 50 μM and a light dose of 30 J/cm^2^ (Er et al., 2015; Ocakoglu et al., 2015b, 2015a).

However, few exceptional studies have been reported contrary to the general principle of diamagnetic and paramagnetic metal complexes of tetrapyrroles. For example, studies in leukemia cells and a rat bladder tumor model have demonstrated that copper octaethylbenzochlorin is an efficient PS despite its nearly undetectable triplet state (Josefsen and Boyle, 2008). The PDT activity of copper octaethylbenzochlorin was attributed to interactions between the cationic iminium group and biomolecules, which allow electron-transfer reactions to take place *via* the short-lived ^1^PS* leading to the formation of radicals and radical ions (Josefsen and Boyle, 2008). Also, Mn complex of meso-tetrakis (carboxyl) porphyrin, Co(II), and Cu(II) methyl pheophorbidea showed significant phototoxicity against cancer cells almost similar to its freebase porphyrin counterpart (Yoon et al., 2011; Shi et al., 2017). Yet another iodinated chlorin p6 copper complex (ICp6–Cu) demonstrated its PDT efficacy against oral cancer cells inducing 90% phototoxicity with 10 μM concentration irradiated with 630 nm at 12 J/cm^2^. Importantly, this complex showed to act predominantly through the type I photochemical process, with the efficacy to induce phototoxicity even under hypoxic conditions (Sarbadhikary et al., 2016).

Other than metals, introduction of halogen atoms in PS also increases ISC through the heavy atom effect and thus can increase the Φ_T_ or τ_T_. For example, fluorinated and chlorinated derivatives of ZnTPP exhibited higher Φ_T_ of 0.99 and 1.02, respectively, compared to Φ_T_ of 0.86 for ZnTPP (Arnaut, 2011). Among the halogenated complexes of 5,10,15,20-tetrakis (4-carbomethoxyphenyl) porphyrin, Φ_T_ reported to be the highest for an iodinated complex (0.88), followed by the brominated complex (0.75), and lowest for non-halogenated PS (0.35) (Marin et al., 2015). These studies showed that halogenation could increase Φ_T_ or τ_T_ of PS, which could significantly influence their efficiency for ^1^O_2_ and other ROS generation. Furthermore, the magnitude of the heavy atom effect has been shown to depend on the type of halogen present within the PS structure. For example, Topkaya et al. (2015) showed that among the four halogen atoms (F, Cl, Br, and I) used for halogenation of trihydroxylated mono-halogenated porphyrin, the ^1^O_2_ quantum yield (Φ_Δ_) was enhanced predominantly by iodination (∼98%).

Another important effect of the insertion of metals in porphyrins, chlorins, and bacteriochlorins is change in absorption and fluorescence properties. In case of porphyrins, insertion of metal causes hypsochromic (blue) shift in the position of the long-wavelength absorption band. For example, the Q band of TPP shifts from 650 nm to 588 nm, 553 nm, and 600 nm for ZnTPP, PdTPP, and InTPP, respectively. Furthermore, the metalation of porphyrins also results in collapse of two out of four Q bands in the spectrum. The later effect is attributed to the increased symmetry (e.g., D2h–D4h) of the tetrapyrrole ring (Dąbrowski et al., 2016). For chlorins, insertion of a metal also causes a similar hypsochromic shift of the long-wavelength Q absorption band without causing any alteration in the symmetry. For chlorophyll derivatives, chlorin e6 and pheophorbide, insertion of Sn^2+^, Pd^2+^, and Cu^2+^ cause ∼15 nm blue shift in the Q band whereas in case of Zn^2+^ the blue shift was only few nm (O’Connor et al., 2009; Yoon et al., 2011; Dąbrowski et al., 2016). The effect is not only observed with the centrally coordinated metal complexes but also observed with ICp6–Cu, where the coordination of Cu^2+^ to the peripheral carboxylic groups resulted in ∼29 nm shift in the Q absorption band at 634 nm from 663 nm for metal-free Cp6 (Sarbadhikary et al., 2016). Ru (II) ions inserted in the porphyrin core have Q absorption bands in the wavelength region between 500–550 nm (Bogoeva et al., 2016). While (TPP) arene–Ru(II) derivatives where arene–ruthenium are inserted as peripheral macrocycle substituents showed no change in Q band position as compared to Q band of TPP at 642 nm (Schmitt et al., 2008). There are some exceptions where insertion of some metal ions causes redshift in the long-wavelength absorption band. For example, insertion of the Sn atom in the central cavity of etiopurpurin, a chlorin PS, causes a redshift of approximately 20–30 nm, with respect to its non-metalated counterpart (Abdel-kader, 2016). Sn (IV) benzochlorin was reported to exhibit an increased photodynamic effect in transplanted urothelial cell carcinoma in rats, as compared to sulphonated benzochlorin (Kessel and Morgan, 1993).

Similarly, the insertion of a metal ion in PS also influences its fluorescence quantum yield (Φ_F_) and lifetime (τ_F_). A general pattern of decrease in fluorescence of tetrapyrrolic compounds is observed for complexes of closed-shell metal, where complexes of first- and second-row elements (e.g., Mg and Al) show longest τ_F_ and higher Φ_F_ than the third-row (e.g., Zn) and fourth-row elements (e.g., Cd and In). Complexes with open-shell central metals such as diamagnetic Pd and paramagnetic Co, Ni, Cu, and Fe have no detectable fluorescence. As per reports, Φ_F_ of TPP, ZnTPP, InTPP, PdTPP, and CuTPP is 0.10, 0.033, 0.05, 0.0002, and 0, respectively, and Φ_F_ for TPPS, ZnTPPS, PdTPPS, and CuTPPS is 0.08, 0.043, <10^−4^, and 0, respectively. The Φ_F_ for Ce6, ZnCe6, and CuCe4 has been reported as 0.13, 0.14, and 0.09, respectively (O’Connor et al., 2009; Dąbrowski et al., 2016). Zn bacteriochlorin exhibits Φ_F_ of 0.13 that is comparable with 0.15 that of its non-metalated PS counterpart. While the Φ_F_ for bacteriochlorins is reduced to 0.02 and 0.006 for its In and Pd complexes, and with no fluorescence for Cu-bacteriochlorin (Chen et al., 2012). The decrease in Φ_F_ is because of the enhancement of internal conversion to the ground state. Paramagnetic complexes have one odd electron that can couple to the spin of the tetrapyrrole ^3^PS* yielding a “tripdoublet” state and a “tripquartet” state. Similarly, that odd electron can couple its spin with that of the ^1^PS*, leading to singmultiplet states. Moreover, singmultiplet states couple efficiently with tripmultiplet states, resulting in manifold increase in ISC from ^1^PS* → ^3^PS*. This coupling mechanism deactivates the ^1^PS* rapidly and quenches almost completely the fluorescence of paramagnetic complexes of tetrapyrroles, while Pd has tightly bound d orbitals that push the intermediate states closer in energy to the ground state, disfavoring the radiationless transition (Arnaut, 2011).

### High-Z PS Conjugates as Chemotoxic and Phototoxic Agents

Tetrapyrrole PS conjugated with either metal complexes and metal ions have been investigated as a promising strategy for selective delivery of toxic metal ions or metallodrug fragment to tumor cells to exploit the advantage of both the chemotoxic and phototoxic effects and/or synergic effect (Boros et al., 2020; Imberti et al., 2020; Otvagin et al., 2022; Smith et al., 2022). For example, in comparison to metal complex such as cisplatin and carboplatin and PS sulfonated pyridinetriphenylporphyrin and hematoporphyrin (Hp) alone or a in combination, conjugates of metal complex PS were shown to exhibit a synergistic antiproliferative effect against various cell lines using both the cytotoxic and phototoxic effects (Brunner and Schellerer, 2003; Kim et al., 2003, 2004; Brunner et al., 2004; Lottner et al., 2004). Furthermore, as compared to carboplatin, certain Pt (II) complexes of Hp derivatives exhibited an elevated tumor-localizing effect (tumor/muscle ratio >2) in tumor-bearing mice (Kim et al., 2003, 2004). In a systemic study, Momekov et al. (2011) reported synthesis of three different types of platinum complexes of Hp IX – 1) The “sitting atop” complex, where Pt (III) is coordinated to two adjacent porphyrin pyrrole nitrogen, 2) the metalloporphyrin-type complex with Pt (III) coordinated through four pyrrole nitrogen of Hp, and 3) the peripheral complex in which Pt (III) is coordinated to the carboxylic groups of propionic acid side chains of Hp. All these complexes showed chemocytotoxic efficacy against various cancer cell lines by targeting DNA; however, complexes with centrally coordinated Pt (III) was found to be more effective than the third complex. Even tetraplatinated porphyrin complexes with peripheral platinum centers were reported to induce synergistic efficacy cisplatin-resistant CP70 carcinoma cell lines. Herein, nuclear localization studies of these complexes and intercalative binding interactions with CT-DNA suggested DNA as the main target for inducing cytotoxicity (Naik et al., 2014).

Further PS with metalloporphyrinates substituted with platinum-based anticancer drugs was evaluated for better water solubility and tumor targeting property. In this study, two pentacationic porphyrinates Ga-4cisPtTPyP (5,10,15,20-tetrakis{cis-diammine-chloro-platinum (II)} (4-pyridyl)-porphyrinato gallium (III) hydroxide tetranitrate) and Ga-4transPtTPyP (5,10,15,20-tetrakis{trans-diammine-chloro-platinum (II)}(4-pyridyl)-porphyrinato gallium (III) hydroxide tetranitrate) were synthesized using the combination of a porphyrin framework, platinum (II)-based groups, and a metal ion gallium (III). Compared to non-Ga 4cisPtTPyP, both the complexes with high Φ_Δ_ of 0.76 and 0.69 for Ga-4cisPtTPyP and Ga-4transPtTPyP, respectively, showed high remarkable photocytotoxicity with significant phototoxic indexes against colon and sarcoma cell lines. Furthermore, Ga-4cisPtTPyP almost completely inhibited tumor growth in an *in vivo* tumor model PDT, with an excellent tumor accumulation capability (tumor/muscle ratio>9) (Hu et al., 2017a). Later, Hu et al. (2017b) reported the synthesis of Zn(II) and In(III) complexes of platinated Pt (II) porphyrins; Zn-4cisPtTPyP and In-4cisPtTPyP showed high Φ_Δ_ of 0.86 and 0.76 compared to non-metaled 4cisPtTPyP of 0.57; however, compared to the Zn complex, the In complex showed excellent *in vivo* phototherapeutic potential on the C26 tumor-bearing mice model, resulting into 75–80% reduction in tumor mass. Another platinum conjugate constructed from tetrakis (4-pyridyl) porphyrin and four oxaliplatin-like moieties induced significant phototoxicity in colon and sarcoma cell lines due to high singlet oxygen generation, nuclear localization, and triggering apoptosis *via* caspase-3–dependent pathway. This complex further showed significant *in vivo* PDT efficacy against colon26 tumor-bearing mice (Hu et al., 2019).

Apart from platinum-based PS, complexes of tetrapyrroles with other metals such as gold (Au) and ruthenium (Ru) have also been reported. Although, tetraphenyl porphyrin-Au (III) complexes showed effective *in vitro* and *in vivo* anticancer chemotoxicity it lacked photosensitizing efficacy (Che et al., 2003; Sun and Che, 2009; Wai-Yin Sun, 2013). Mechanistically, one of these Au(III) porphyrin induced cell death by targeting mitochondrial membrane potential, resulting into apoptosis through both caspase-dependent and caspase-independent mitochondrial pathways (Wang et al., 2005). Similarly, Au(II) complex of Hp showed chemocytotoxic efficacy by inducing DNA fragmentation in several leukemia- and lymphoma-derived tumor cell lines. Interestingly, as a proof of selective killing, Au(II)–Hp showed less cytotoxicity against non-cancerous human kidney cell line 293T in comparison to cisplatin, which induced similar cytotoxic effects both in non-cancerous and cancer cell lines (Momekov et al., 2008). Grin et al. (2019) reported the synthesis of a conjugate of a pro-oxidant thiolate Au (I) moiety with the bacteriopurpurin core. Most importantly, bacteriopurpurinimide with far red absorption property (λ ∼ 800 nm) can be applied for deep tissue irradiation. Au thiolate is supposed to inactivate glutathione reductase thus have cytotoxic efficacy even without light irradiation. *In vitro* studies against HCT116 cells showed markedly high cytotoxicity in the dark alongside with high phototoxic efficacy, due to the increase of the ROS level in the dark, mediated by glutathione reductase breakdown. Furthermore, the *in vivo* studies in the S37 sarcoma cells–transplanted mice model showed high tumor-to-skin ratio of ∼2.5 and partially inhibited the tumor growth in the dark, while PDT of mice completely blocked the tumor growth (Grin et al., 2019).

As Ru–porphyrin complexes, nitrogen atoms coordinated Ru metal ion porphyrin complexes and a polypyridyl–Ru(II) complex conjugated to macrocycle as peripheral substituents have been reported (Bogoeva et al., 2016). While porphyrin–Ru(II) polypyridyl conjugate showed both chemotoxicity and phototoxicity; the complexes having Ru coordinated with nitrogen atoms failed to show any anticancer activity (Hartmann et al., 1997; Gianferrara et al., 2010). Another study reported an extended-arm Ru–porphyrins with potential chemotoxic and phototoxic anticancer effects against human breast cancer cells. Importantly, as compared to MDA-MB-231 cancer cells, the chemocytotoxic effect of these complexes was significantly less in non-tumorigenic epithelial HBL-100 cells (Gianferrara et al., 2010). Other examples of Ru-PS complexes with both phototoxic and cytotoxic potentials include Ru–porphyrin conjugates bearing four peripheral Ru(II) half-sandwich coordination compounds and centrally inserted Ru(II)–porphyrin (Schmitt et al., 2008; Bogoeva et al., 2016).

In addition to these Ru, Pt, and Au tetrapyrrole complexes, Cu (II) complex of (5,10,15-tripyridyl-4-yl-porphyrin- 5-yl) benzonitrile has also been reported to exert chemo and photocytotoxicity against the human breast cancer cells, wherein the dark toxicity of this compound was shown to be mediated through ROS generation (Antoni et al., 2015). Similarly, another Cu-PS complex, that is, ICp6-Cu besides its phototoxic efficacy showed pronounced chemocytotoxicity in oral cancer cells through elevation of intracellular ROS; however, without affecting the viability of the non-cancerous keratinocyte cells. Interestingly, an induced cell death mechanism was reported to be other than *via* necrosis or apoptosis, with highly vacuolated cytoplasm, permeabilization of lysosomal membrane, and damaged cytoskeleton F-actin filaments (Sarbadhikary and Dube, 2017b).

**FIGURE 5:**
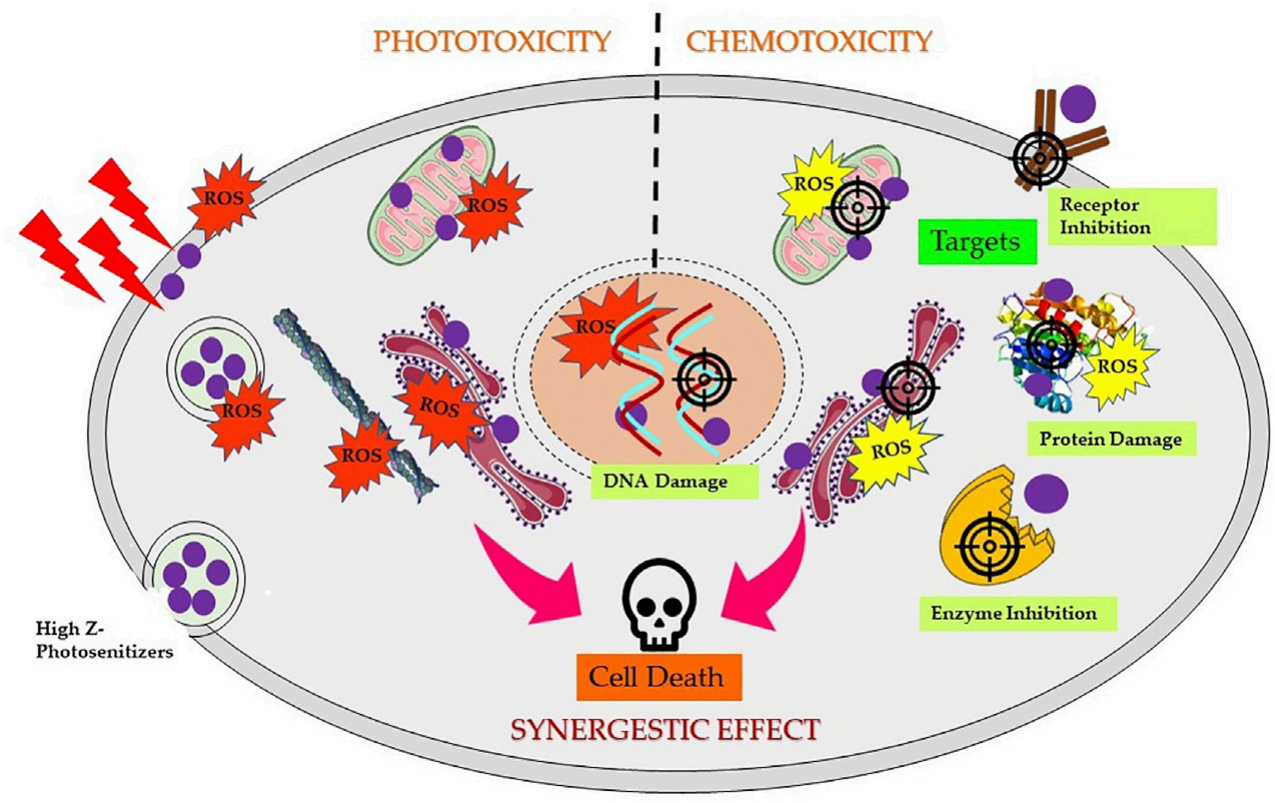
Scheme of mechanism of synergistic effects induced by high Z element-based photosensitizers triggering cancer cell death. Depending on the localization of complexes in different subcellular compartments and followed by PS excitation by appropriate wavelength of light generates reactive oxygen species (ROS), resulting into direct or indirect photodynamic damage to the endoplasmic reticulum, lysosome, mitochondria, and/or plasma membrane. Chemotoxic PS complexes target and interact with specific cellular targets such as DNA, receptors, enzymes, proteins, or mitochondria, resulting in DNA damage, ROS generation, and inhibition and/or damage of several vital targets in dark and activate several cell death pathways.

5,10,15,20-Tetra(4-N-allylpyridyl) porphine tetrabromide and 5-mono (3′-methoxy-4′-hexadecyloxyphenyl)-10,15,20-tri (4N-allylpyridyl) porphine tribromide complexed with Ag have also shown promising chemotherapeutic activity against cancer cells (Tovmasyan et al., 2008, 2014). Furthermore, Pd(III) complexes of Hp reported to induce chemotoxicity *via* DNA fragmentation against a range of cancer cells (Tsekova et al., 2013). Figure 5 shows schematic mechanistic insights for synergistic effects *via* both induced phototoxicity and chemotoxicity.

### High-Z PS Conjugates for X-Ray Photon Activation Therapy

In 2006, Chen and Zhang proposed a new treatment strategy by exploiting the deep tissue penetration potential of X-ray radiation for photoactivation of PS. This strategy holds a great potential to treat deep-seated tumors by overcoming the limitation of low treatment depth in PDT. Moreover, that combination of conventional radiation therapy with PDT lowers down the radiation doses, thus preventing collateral damage to surrounding tissues. An X-ray–combined PDT approach makes use of PS conjugated to lanthanide or metal scintillating nanoparticles, which by the process of scintillation converts X-rays to UV/visible light to photoactivate the attached PS (Chen and Zhang, 2006; Morgan et al., 2009; Kamkaew et al., 2016). However, the efficacy of this approach depends on several factors such as effective energy transfer between PS and scintillating material, cellular uptake of the conjugate, and most importantly on Φ_Δ_, which in deeper tumor region will be limited by hypoxic conditions (Huang et al., 2008; Morgan et al., 2009; van Straten et al., 2017).

**FIGURE 6:**
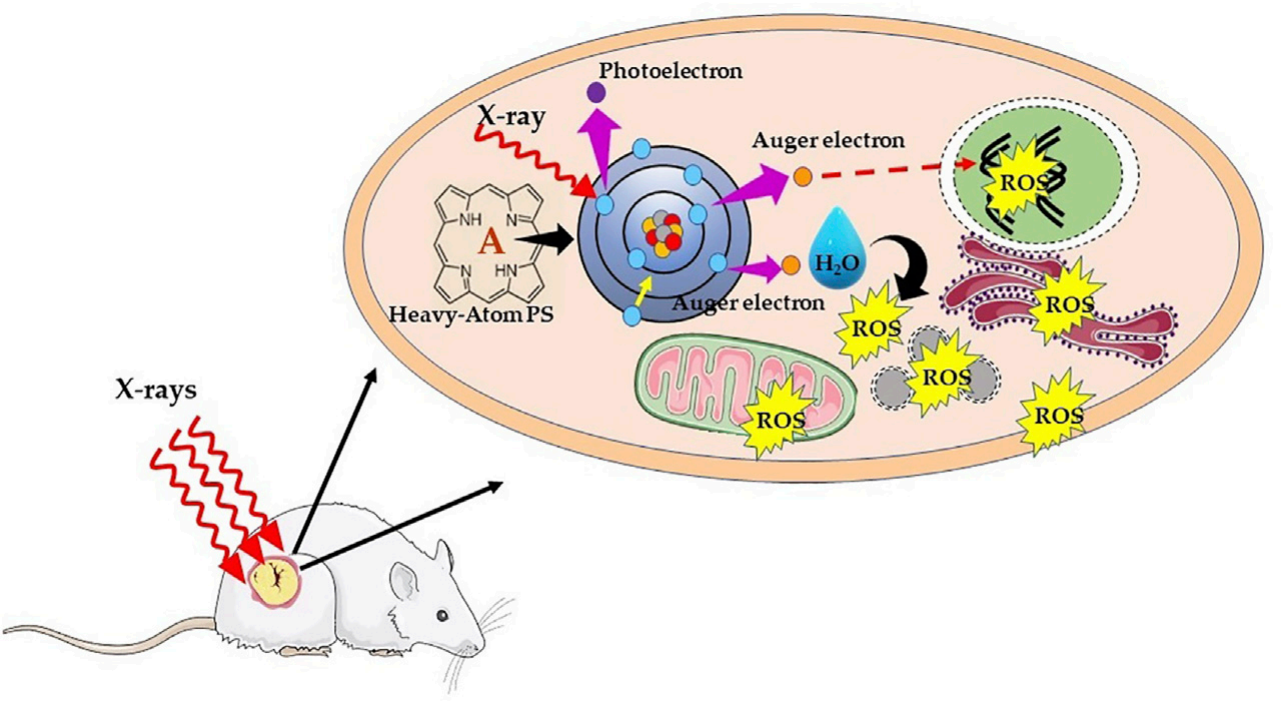
Schematic illustration of mechanism of X-ray photon activation therapy. Interaction of X-rays with an inner shell electron of heavy atom or metal causes the ejection of the electron, that is, photoelectron. Later, an electron of an outer shell (higher energy state) drops into the lower energy shell to fill the vacant electron. The energy difference between the two shells is then transferred to another electron, which is ejected from an outer shell as Auger electron with an energy of ∼20–500 eV and shorter range (<1 μm). The successive ejections of further Auger electrons leading into an Auger cascade causing direct DNA damage, and/or result in water hydrolysis and production of ROS, which indirectly leads to DNA damage.

Alternatively, another chemoradiotherapy strategy proposed by Fairchild et al. make use of X-ray absorbing metal-based compounds having primarily to enhance X-ray dose deposition in tumor cells (Laster et al., 1993). This forms the principle of photon activation therapy (PAT), whereby direct absorption of X-rays by metalated PS subsequently generates Auger electrons and free radicals using the photoelectric effect. The drug candidates for PAT contain high Z atom (Pt, Au, I, or Br) and generally designed with the purpose to target the nucleus in tumor cells. The tumor mass is then irradiated with X-ray of energy slightly above the K-edge absorption of the metal or halogens. As represented schematically in Figure 6, the mechanism in photon-activation of high-Z atoms leads to the emission of Auger electrons and photoelectrons, which in turn reacts with nearby biomolecules or induces the formation of free radicals through radiolysis of water to cause direct or indirect cellular damage, a phenomenon known as the photoelectric effect. Thus, the photon absorption by high-Z atoms contributes to the enhancement of energy deposition such that efficient tumor damage can be achieved even with low irradiation dose (Fairchild et al., 1982; Laster et al., 1993; Kobayashi et al., 2010; Gil et al., 2011). Furthermore, free radical generation *via* Auger electrons is less dependent on O_2_ concentration, which in turn suggests that it is less likely that PAT will be affected by hypoxia prevailing in solid tumors.

Several studies have proved the biological effectiveness of PAT. For example, monochromatic synchrotron X-ray irradiation of HeLa and Chinese hamster ovary (CHO) cells treated with 5-bromodeoxyuridine radiation induced significantly higher cell death when irradiated with X-ray energy at the K-absorption edge (∼12.40 keV) of Br than well above K-edge (13.78 keV) (Shinohara et al, 1985). Similarly, Larson et al. (1989)showed as compared to radiation energy below K-edge, X-ray irradiation above the K-edge absorption of Br increased the radiosensitization effect by ∼ 3–12% in bromodeoxyuridine BrdU–treated Chinese hamster V79 cells. Also, studies at the National Synchrotron Light Source of Brookhaven National Laboratory revealed the prominent radiosensitization effect and cell killing by synchrotron X-ray irradiation on iododeoxyuridine IUdr–treated Chinese hamster V79 cells with monochromatic photons at iodine K-edge (33.4 keV) than below its energy (32.9 keV) (Laster et al., 1993).

Currently, platinated drugs (cisplatin and carboplatin) and iodinated compounds in combination with X-ray are actively being investigated for the treatment of radio-resistant brain cancer (Biston et al., 2004; Adam et al., 2006, 2008; Rousseau et al., 2009; Ricard et al., 2013). The underlying reason being the induced radiosensitization effect by metal complexes results in higher dose deposition in tumor tissue and since a relatively low dose of radiation is required, which can effectively reduce collateral damage to the surrounding normal tissues. This makes PAT a promising alternative treatment approach for the treatment of high-grade brain tumors with a promising outcome. In a systemic study, Ceresa et al. (2014) reported that cisplatin treatment induced substantially higher cell killing upon irradiation with synchrotron X-ray produce compared to conventional X-ray irradiation in highly resistant glioblastoma multiforme cells. Furthermore, studies in glioma-bearing rats and mice showed that administration of iodine or platinum compounds and its activation with X-ray demonstrated higher survival compared to radiation alone (Biston et al., 2004; Adam et al., 2008; Rousseau et al., 2009; Ricard et al., 2013; Ceresa et al., 2014). However, the use of cisplatin is associated with severe side effects, which warrant development of less toxic and more effective agents to fully exploit the advantages of PAT as a potential therapeutic approach (Astolfi et al., 2013). PS conjugated to high-Z elements are promising alternative candidate for PAT, as PS further provides with the benefit of preferential tumor accumulation. However, except few agents, such as Au complex of chlorin e6 (Tsuchida et al., 2003), iodinated pyropheophorbide derivatives (Ishibashi et al., 2013), and ICp6-Cu (Sarbadhikary and Dube, 2017a), the efficacy of PS for X-ray PAT of cancer is not well investigated.

Mono-L-aspartyl aurochlorin e6 (Au-NPe6) was investigated for diagnosis and treatment of tumor by synchrotron X-ray radiation whereby tumor-bearing mice treated with Au-NPe6 showed good absorption contrast on X-ray films. Moreover, X-ray irradiation of Au-NPe6 pretreated mice with 25 keV synchrotron radiation showed a slow rate of tumor growth as compared to untreated mice (Tsuchida et al., 2003). Furthermore, the studies carried out by Pandey et al. reported iodinated HPPH (I-HPPH) as a selective radiosensitizer for tumor when irradiated with K-edge X-ray energy of 33 keV for iodine. I-HPPH exhibited a significant *in vitro* radiosensitization effect in treated human bladder cancer cells caused by a significant increase in ROS generation, following X-ray irradiation. Mechanistically, the treatment induced mitochondria-mediated radiosensitized cell death, due to the localization of I-HPPH in mitochondria. Furthermore, the *in vivo* studies showed X-ray irradiation of I-HPPH pretreated tumor-bearing mice showed a delayed growth rate of tumor as compared to mice treated with X-ray alone (Ishibashi et al., 2013).

Similarly, photodynamically active ICp6-Cu was reported to exhibit anticancer radio-sensitization efficacy in two human oral cancer cell lines in combination with synchrotron X-ray radiation (8–10 keV), with sensitization enhancement ratios of 1.8 and 2.8, for concentrations of 20 and 30 μM, respectively. The radiosensitization effect of ICp6-Cu accompanied a significant increase in the ROS level, lysosomal damage, and inhibition of repair of radiation-induced DNA double-strand breaks, eventually leading to increase in cell death as compared to X-ray alone effects (Sarbadhikary and Dube, 2017a).

### Toxicity and Safety Issues Associated With Inorganic Complexes of PS

Undoubtedly, the role and importance of high Z-based PS complexes have tremendously revolutionized the field of cancer therapy and diagnosis. However, *in vivo* applications of inorganic compounds with metals and heavy elements also arouse the concern for their long-term and systemic toxicity in the body as well as with the clearance. Moreover, the prediction of toxicity issues is relatively difficult to understand as it depends on several different factors 1) physiologically essential and non-essential elements and 2) route of administration, solubility, oxidation state, bioavailability, redox property, and kinetic stability of the complexes, and 3) their *in vivo* pharmacodynamics and pharmacokinetic properties, which differs greatly from their pure elements (Egorova and Ananikov, 2017). Thus, understanding the behavior, possible interactions, and reactivity of high Z element containing PS complexes in real biological systems is crucial for the safe implementation of these complexes and before proceeding into clinical applications. Until now, many strategies are being explored to prevent or limit the associated toxic effects of potential complexes, which include encapsulation within biocompatible nanoarchitectures and/or bio-inspired delivery mechanisms (Stacey and Pope, 2013; Renfrew, 2014; Długosz et al., 2020). Moreover, the arena of medicinal chemistry research has advanced markedly from the rather crude “synthesis and cytotoxicity screening” approach to a whole toolbox of modern biomedical research, combining diverse fields starting from conventional biochemical and cell-based assays to advanced structural biology and computer-aided designing. Such strategies will certainly allow to gain deep insights into possible cellular and molecular details of complex interactions before proceeding into *in vivo* testing and bring at least some of the most promising drug candidates to the market (Gasser et al., 2011).

## Conclusion

PDT has already demonstrated its potential as a single or combination therapy in clinics for the treatment of cancer. However, as discussed, PDT and PS suffer from several limitations. Among the several novel strategies to improve the PDT outcome, incorporation of high-Z metals/halogens has gained attention to overcome the drawbacks of present clinically approved PS. In this review, we attempted to provide the general designing strategies of high-Z-element-based PS, which includes introduction of transition metals and halogen as central insertion or side group substitution in the tetrapyrrolic structure. The introduction of heavy atoms offers several advantages of enhancing triplet excited state lifetime and ^1^O_2_ generation, and improving deeper tissue penetration by redshift in the absorption spectra of PS. The unique photophysical and photochemical properties imparted by a wide array of different heavy atoms offer the potential of choosing and tuning the overall photodynamic efficacy by choosing from the broad spectrum of high Z elements. As shown in Table 1, several metals containing PS are either clinically approved or already being investigated in clinical trials in different countries worldwide. In addition to their promising photophysical and photodynamic potentials, high-Z-atom PS also offer the advantages as multitherapeutic drug candidates for combinational anticancer therapy.

**TABLE 1:** List of metal or heavy atom-based photosensitizers approved or in clinical trials for photodynamic therapy of cancer

Generic name	Chemical name	Wavelength (λ_max_)	Clinical status
TLD-1433	Ruthenium–polypyridine	525 nm	In clinical trials, non-muscle invasive bladder cancer
Purlytin	Tin ethyl etiopurpurin	664 nm	In clinical trials, basal cell cancer, Kaposi’s sarcoma, prostate cancer, and breast adenocarcinoma
	Silicon phthalocyanine	675 nm	In clinical trials, skin cancer
Photosens	Aluminum phthalocyanine tetrasulfonate chloride	676 nm	In clinical trials, stomach, skin, lip, oral, and breast cancers
	Zinc phthalocyanine	676 nm	In clinical trials, skin cancer and squamous cell carcinoma
Lutrin	Motexafin lutetium	732 nm	In clinical trials, prostate, breast, cervical, brain, skin, and superficial cancers
Stakel	Palladium-bacteriopheophorbide (WST11)	∼750 nm	In clinical trials, prostate cancer
TOOKAD soluble	Palladium-bacteriopheophorbide (WST09)	763 nm	**Approved** prostate cancer

Despite their several advantages, high-Z PS suffer from low water solubility and serious aggregation-induced quenching (ACQ) effects in aqueous media as well as poor tumor selectivity and heavy metal or atom induced short- or long-term *in vivo* toxicity. Furthermore, several different and potential designing strategies are needed to be explored, involving water-soluble and non-aggregating PS, long wavelength absorbing PS, employing two-photon excitation and introduction of targeting moieties, nanodelivery systems, “one-for-all” or “all-in-one” and activable heavy atom PS to improve tumor imaging and therapeutic efficacy. Thus, all these warrants serious investigations in future work to assess the full potential of heavy atom PS, before proceeding to clinical translation.
